# Measuring trust in public health authorities in the United States: An item response theory analysis

**DOI:** 10.1371/journal.pone.0355498

**Published:** 2026-08-06

**Authors:** Samantha Kloft, Raquel Guimaraes, Jung-Hee Hyun, Daniel López-Cevallos

**Affiliations:** 1 Department of Health Promotion and Policy, University of Massachusetts, Amherst, Massachusetts, United States of America; 2 International Institute for Applied Systems Analysis, Laxenburg, Austria; University of Zagreb School of Medicine: Sveuciliste u Zagrebu Medicinski fakultet, CROATIA

## Abstract

Trust in public health authorities is central to effective public health governance, shaping communication, policy implementation, and collective action. Despite its importance, trust has been measured using a wide range of approaches, and comparatively few studies rely on validated instruments, limiting comparability across studies and contexts. This study applies Item Response Theory (IRT) to refine the beneficence dimension of the Trust in Public Health Authorities (TiPHA) Scale, originally developed and validated by Holroyd et al. (2021) using confirmatory factor analysis. Using cross-sectional, nationally representative data from the 2024 RAND American Life Panel Omnibus Wave 17 survey (n = 2,034), we evaluated the dimensionality and item-level performance of the eight-item TiPHA beneficence subscale. A graded response model was estimated to assess item discrimination, threshold ordering, and information. Results supported unidimensionality and high internal consistency for the full subscale (α = 0.88), but revealed substantial variation in item performance. Three negatively worded items demonstrating very low discrimination and minimal information were removed. The resulting five-item scale retained robust reliability (α = 0.88) and exhibited stronger measurement coherence, providing greater precision at moderate to high levels of trust. Posterior latent trait estimates were generated and rescaled to a 0–10 metric for interpretability. When applied descriptively across sociodemographic and socioeconomic characteristics, the refined trust score exhibited modest but interpretable variation, with higher average trust observed among older adults and individuals with higher educational attainment, consistent with prior theoretical expectations. These patterns support the scale’s use as a population-based measure of trust in public health authorities. Future research should apply this refined scale in multivariable and intersectional analyses to examine whether observed differences persist after accounting for correlated social, economic, and structural factors.

## Introduction

Trust in public health authorities is a foundational component of public health governance, shaping the capacity of institutions to communicate risk, implement policy, and coordinate collective action [1–4]. Because public health authorities operate primarily through guidance, regulation, and interventions, their effectiveness depends not only on technical capacity but also on public confidence in their intent and legitimacy. When trust is present, public health institutions are better positioned to function as credible stewards of population health [5,6]; when it is absent, even well-designed interventions may fail to achieve their intended impact [7–10].

Despite its importance, trust in public health authorities has been measured inconsistently across studies [11,12]. Quantitative surveys often rely on single or unvalidated items, and even validated tools differ in their focus on specific authorities, trust dimensions, or phrasing, limiting their comparability [11–14]. The Trust in Public Health Authorities (TiPHA) Scale, developed by Holroyd and colleagues (2021), advanced the field by offering a psychometrically validated instrument capturing two dimensions of trust: beneficence and competence [13]. Initial validation of the TiPHA Scale relied on confirmatory factor analysis to establish its dimensional structure and internal consistency, providing a useful first step in scale development but offering limited insight into item-level measurement properties [15].

A key challenge in trust research is that it is a latent construct, meaning it cannot be directly measured and must be estimated before it can inform empirical studies or practical applications. Item Response Theory (IRT) offers a sophisticated framework for modeling such latent traits at the item level, providing more theoretically grounded and detailed measurement than traditional approaches such as factor analysis [16,17]. IRT has long been applied in psychological and educational testing [18], but its use has since expanded. Recent applications include health measurement more broadly [19,20], clinical assessment [21], and even scales for environmental risk perception [22]. This broader use demonstrates the adaptability of IRT in capturing attitudes, perceptions, and other constructs that, like trust, cannot be directly observed.

In this study, we applied IRT to further refine the TiPHA beneficence subscale using data from the nationally representative RAND American Life Panel (ALP) Omnibus Wave 17 survey [23]. The study pursued three aims: (1) to assess the dimensionality and item performance of the eight-item TiPHA beneficence subscale; (2) to develop a revised, psychometrically robust trust score with improved measurement efficiency and interpretability; and (3) to descriptively apply the refined trust score across key sociodemographic and socioeconomic characteristics. By evaluating item-level discrimination, threshold ordering, and information, this study identifies the most effective indicators of trust in public health authorities and strengthens the methodological foundation for using concise, reliable measures in large population surveys.

## Materials and methods

### Data source

This study employed a psychometric scale refinement design using cross-sectional data from RAND’s 2024 Omnibus Wave 17 survey. Analyses were conducted using the full sample of 2,034 respondents to evaluate item performance, estimate respondent-level latent trait scores, and examine the distribution of the refined trust measure. All analyses were conducted in Stata version 18, with probability weights incorporated into IRT model estimation and applied to descriptive analyses to support national representativeness. This secondary analysis of de-identified data was determined exempt by the University of Massachusetts Amherst Institutional Review Board.

### Study population

The Omnibus Wave 17 survey was fielded from February to March 2024 with a sample of 2,034 adults recruited through RAND’s ALP using probability-based sampling. Eligibility was limited to US residents aged 18 and older; in this wave, respondents ranged from 20 to 90 years old. The ALP is a longitudinal survey panel in which participants may remain enrolled across multiple survey waves, contributing to relatively low attrition over time. Omnibus surveys typically achieve completion rates of approximately 70–80%, and respondent demographic characteristics are updated regularly through a household survey patterned after the Current Population Survey. Detailed descriptions of RAND’s recruitment, weighting, and retention procedures have been published previously [24]. Key demographic characteristics of the sample are presented in Table 1, with full details provided in Table S1 of the S1 File.

**Table 1:** Key demographic characteristics of survey respondents (N = 2,034).

Characteristic	n (%)
Sex	
Female	1,041 (51.2%)
Male	993 (48.8%)
Age	
20–34	559 (27.5%)
35–54	690 (33.9%)
55–74	645 (31.7%)
75+	140 (6.9%)
Race	
White/Caucasian	1,530 (75.2%)
Black/African American	256 (12.6%)
American Indian/Alaska Native	24 (1.2%)
Asian or Pacific Islander	61 (3.0%)
Other (Unspecified)	163 (8.0%)
Ethnicity	
Hispanic/Latinx	384 (18.9%)
Non-Hispanic/Latinx	1,650 (81.1%)
Residence Region	
Northeast	417 (20.5%)
Midwest	323 (15.9%)
South	840 (41.3%)
West	454 (22.4%)
Education	
High School or Less	777 (38.2%)
Some College/Associate Degree	535 (26.3%)
Bachelor’s Degree	399 (19.6%)
Graduate Degree	323 (15.9%)
Family Income	
Low (<$25k)	252 (12.4%)
Lower–Middle ($25k–$49,999)	399 (19.6%)
Middle ($50k–$99,999)	635 (31.2%)
Upper–Middle ($100k–$199,999)	618 (30.4%)
High (≥$200k)	130 (6.4%)

### Measurement tools

Trust in public health authorities was assessed using items from the Trust in Public Health Authorities (TiPHA) Scale, originally developed and validated by Holroyd and colleagues (2021) [13]. The full instrument comprises 14 items across two distinct dimensions of trust. The *beneficence* dimension captures perceptions that public health authorities act ethically, fairly, and in the public’s best interests, whereas *competence* reflects perceptions of technical expertise and capacity [11,13]. Initial validation conducted by Holroyd and colleagues using confirmatory factor analysis supported this two-factor structure, with high internal consistency reported for both beneficence (Cronbach’s α = 0.92) and competence (Cronbach’s α = 0.87).

Due to space limitations, the Omnibus Wave 17 survey included only the eight-item beneficence subscale, which Holroyd and colleagues found to be a strong predictor of overall trust in public health authorities [13]. Beneficence is particularly well suited for psychometric refinement because it captures normative evaluations of institutional intent and ethical commitment that are central to how trust is formed and expressed in public health contexts. These evaluations are inherently subjective and continuous, making them especially sensitive to item wording, scale structure, and measurement precision. Improving measurement of beneficence therefore has important implications for how trust in public health authorities is assessed and compared across populations and studies.

Items in the Omnibus Wave 17 survey were administered using a six-point Likert-scale ranging from “Strongly Disagree” to “Strongly Agree,” with an additional “Not Applicable/Don’t Know” response option. Responses of “Not Applicable/Don’t Know” were treated as missing and excluded from analyses. Item-level missingness was low, ranging from 1.4% to 3.9% across the eight beneficence items (survey-weighted range: 1.4%−3.7%; Table S2 of the S1 File). The majority of respondents provided complete data, with 89.9% answering all eight items. A small number of respondents (n = 6; 0.3%) provided no valid responses on any trust item and were excluded from IRT estimation. Three items were negatively worded in the original scale (items 2, 5, and 7) and were reverse-coded prior to analysis to ensure consistent directionality before item-level evaluation. Full item wording is presented in Table 2.

**Table 2:** TiPHA Scale Questions on Beneficence utilized in the 2024 Omnibus Wave 17 Survey. Please indicate your level of agreement with the following statements about public health authorities, such as local and state health departments, the Centers for Disease Control and Prevention (CDC) and Food and Drug Administration (FDA).

1. They do everything they should to protect the health of the public.
2. They keep trying the same things to help the public, even when they don’t work very well.*
3. They base recommendations on the best available science.
4. They are concerned about all people, without caring about who has more or less money.
5. They are concerned about some racial and ethnic groups more than others.*
6. They accurately inform the public of both health risks and benefits of medicines.
7. They make unhelpful recommendations.*
8. They believe in what they recommend for the public.

* Negatively worded items were reverse coded prior to analysis to ensure comparability.

In addition to the trust scale, the survey collected a standard set of sociodemographic variables, including sex, age, race, nativity, residence region, education, income, employment status, and insurance coverage. A complete list of covariates is provided in Table S1 of the S1 File.

### Analytic approach

To address the first study aim, dimensionality of the eight beneficence items was evaluated to assess their suitability for IRT modeling. Essential unidimensionality was examined using a weighted polychoric correlation matrix and principal components analysis (PCA), and internal consistency was assessed using Cronbach’s alpha. These diagnostics were used to confirm that the beneficence items reflected a single latent construct appropriate for IRT-based scale refinement.

Building on this assessment, IRT was applied to model trust as a latent trait and to estimate item-level parameters describing how individual items function across levels of trust [25,26]. By modeling each item’s relationship to the underlying latent construct, IRT allows assessment of item discrimination, threshold ordering, and measurement precision across the trait continuum [27]. Item information functions were used to identify poorly performing items and to guide refinement of the scale toward a concise yet psychometrically robust measure [25].

Specifically, a graded response model (GRM) [28] was estimated using the full survey sample. The GRM assumes ordered response categories and a monotonic relationship between the latent trait and the probability of endorsing higher response options. Item-level parameters were examined to evaluate performance: discrimination parameters assessed how well each item differentiated respondents across levels of trust, while threshold parameters indicated the level of the latent trait required to endorse each successive response category.

Item characteristic curves (ICCs), which depict the probability of selecting each response option across levels of trust, and item information functions (IIFs), which quantify measurement precision across the latent continuum, were examined to identify poorly performing items. Items demonstrating low discrimination or poorly ordered thresholds were excluded, and the GRM was re-estimated using the retained items. The dimensionality and internal consistency of the refined scale were reassessed using the polychoric correlation matrix, PCA, and Cronbach’s alpha. Finally, a test information function (TIF), summarizing overall measurement precision across levels of trust, was examined to evaluate the performance of the shortened scale.

To address the second study aim, posterior latent trait estimates (θ) were generated for each respondent based on the final GRM specification. These estimates were linearly rescaled to range from 0 to 10 for interpretability, with higher values indicating greater trust in public health authorities.

To address the third study aim, the resulting IRT-based trust score was descriptively examined across sociodemographic and socioeconomic subgroups using survey-weighted summary statistics, including means, standard errors, and 95% confidence intervals, as well as distributional analyses of score range and density. This application was intended to evaluate the scale’s substantive behavior within the full survey sample rather than to test causal or explanatory hypotheses. Item parameters, scoring procedures, and diagnostic results are reported transparently to facilitate replication and support reuse of the refined measure in future research.

## Results

### Item response theory analysis

**Fig 1:**
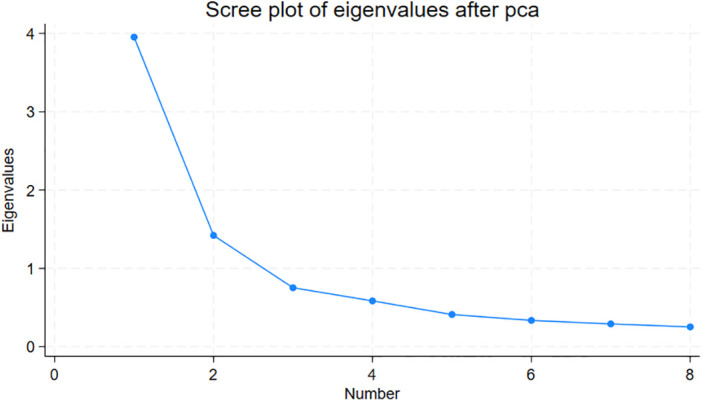
Scree plot of the 8-item TiPHA beneficence scale.

Subscale items supported a unidimensional structure, with the first eigenvalue estimated through PCA substantially larger than the second. The scree plot in Fig 1 shows a clear drop after the first component, confirming that the items captured a single underlying construct. As shown in Table S3 of the S1 File, the polychoric correlation matrix indicated uniformly positive associations among items, with most correlations in the moderate-to-strong range (≈0.65–0.78) and lower values for the three reverse-coded items (≈0.15–0.43). This pattern further supports a common underlying construct, consistent with the PCA results. Internal consistency was high (Cronbach’s α = 0.88), suggesting strong reliability for the full 8-item scale.

**Fig 2:**
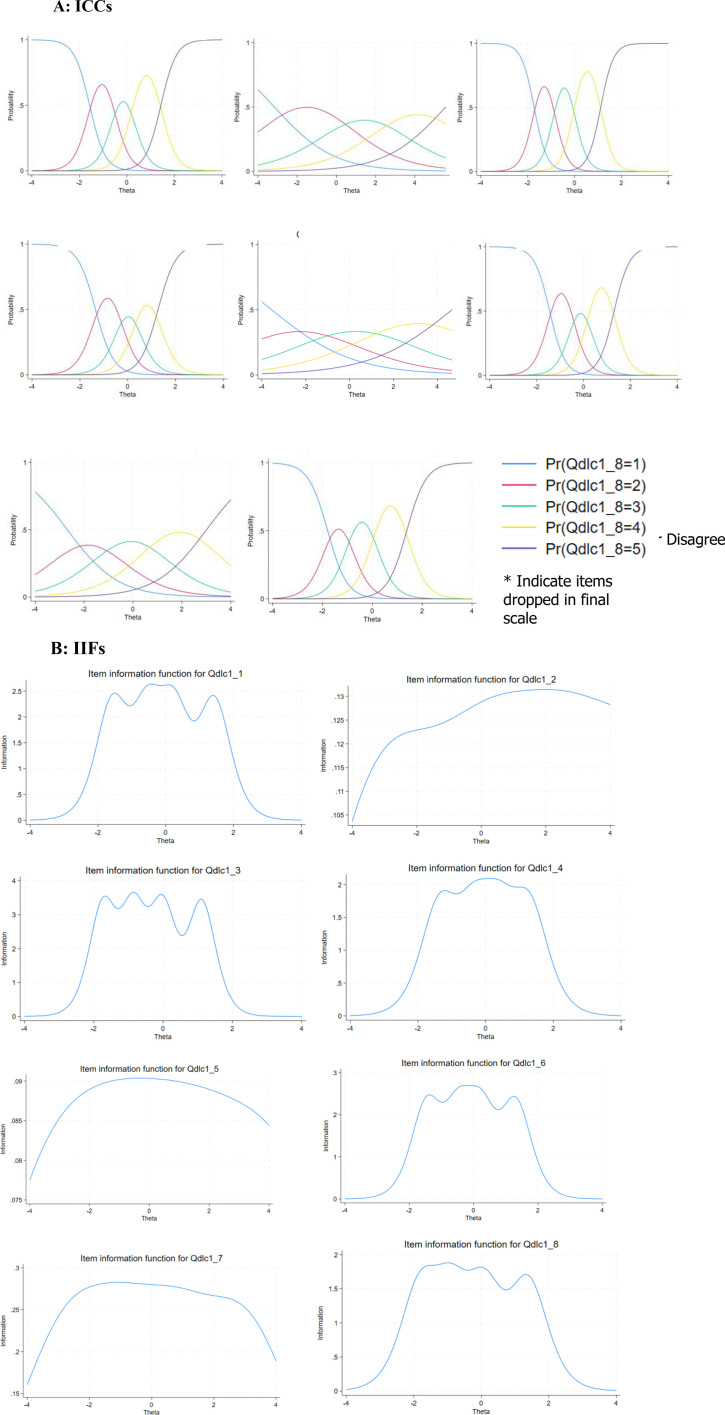
ICCs and IIFs for the 8-item TiPHA beneficence scale.

However, the initial IRT GRM highlighted clear differences in item performance. Table 3 presents the discrimination and threshold parameters for all items. Items 1, 3, and 6 demonstrated very strong discrimination (a ≈ 2.7–4.0) and contributed the most information, particularly at moderate to high levels of trust. Items 4 and 8 also performed well, with information peaks around 2.0, which is generally considered a threshold for good measurement precision. By contrast, the reverse-coded items 2, 5, and 7 showed very low discrimination (a < 0.5) and provided little precision across the latent continuum. This pattern is illustrated in the ICCs and IIFs in Fig 2, where the weaker items display flat curves and minimal information compared with the steep slopes and higher peaks of the stronger items.

**Table 3:** Item parameters for the 8-item TiPHA beneficence scale.

Item	Discrimination (a)	Threshold 1 (b1)	Threshold 2 (b2)	Threshold 3 (b3)	Threshold 4 (b4)
1	3.07	−1.57	−0.54	0.23	1.43
2*	0.66	−3.16	0.15	2.70	5.57
3	3.69	−1.72	−0.86	−0.02	1.11
4	2.67	−1.35	−0.34	0.38	1.27
5*	0.54	−3.56	−0.99	1.58	4.67
6	3.07	−1.45	−0.47	0.22	1.30
7*	0.96	−2.68	−0.99	0.83	3.00
8	2.57	−1.79	−0.91	0.08	1.38

*Note:* Negatively worded items (*) were reverse coded prior to analysis. Parameters estimated using a graded response model with survey weights. Discrimination (a) reflects item slopes; thresholds (b1-b4) represent locations on the latent trait (trust in public health authorities) where adjacent response categories are equally probable.

**Fig 3:**
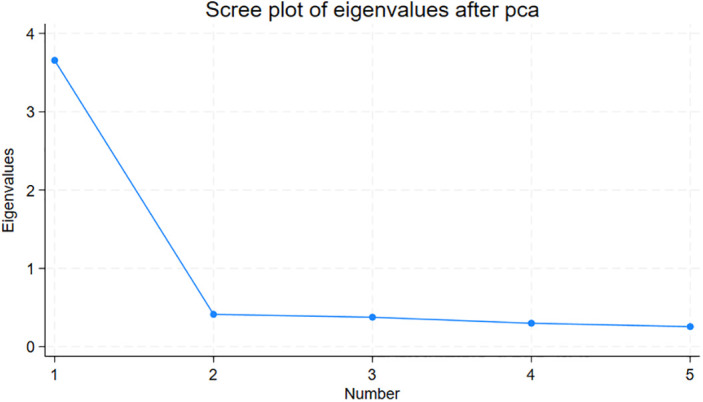
Scree plot of the refined 5-item TiPHA scale.

**Fig 4:**
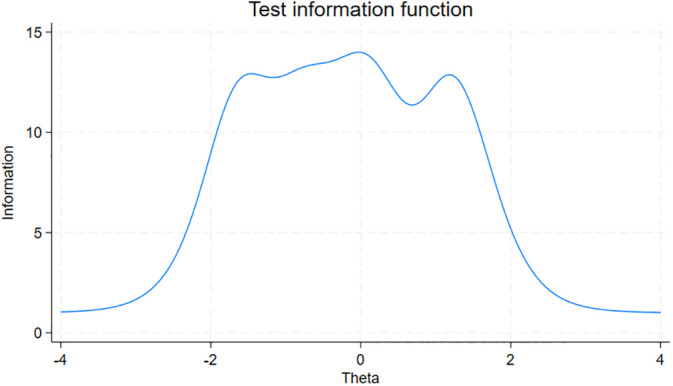
Test information function for the refined 5-item TiPHA scale.

Based on these results, and guided by recommendations from the literature, items 2, 5, and 7 were excluded, yielding a refined 5-item scale [29]. Table 4 reports the estimated parameters for the five retained items, all of which showed strong discrimination (a = 2.56–3.69) and well-ordered thresholds. Cronbach’s α for the shortened 5-item scale remained high (0.88), comparable to the 8-item version (indeed, the scree plot in Fig 3 again supports unidimensionality). The polychoric correlation matrix for the refined items also showed higher and more consistent magnitudes than in the full scale, further reinforcing the coherence of the construct. The TIF for the refined model, displayed in Fig 4, indicates that the scale provided the greatest measurement precision for respondents with moderate to high trust (θ ≈ −0.5 to +1.5), with declining reliability at the lowest levels of trust.

**Table 4:** Item parameters for the refined 5-item TiPHA beneficence scale.

Item	Discrimination (a)	Threshold 1 (b1)	Threshold 2 (b2)	Threshold 3 (b3)	Threshold 4 (b4)
1	3.12	−1.56	−0.52	0.24	1.41
3	3.69	−1.74	−0.85	−0.01	1.11
4	2.69	−1.34	−0.33	0.38	1.26
6	3.12	−1.43	−0.45	0.22	1.29
8	2.56	−1.80	−0.90	0.09	1.38

*Note:* All items showed strong discrimination (a > 2.5), indicating they effectively differentiate respondents across levels of trust in public health authorities. Thresholds represent points on the latent continuum where adjacent response categories are equally likely. Parameters estimated using a graded response model with survey weights applied.

### Application of refined trust scale

**Fig 5:**
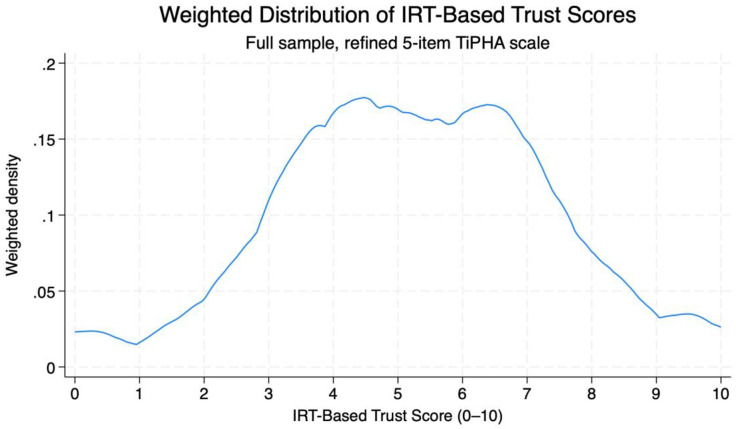
Distribution of the refined IRT-based trust score (0-10).

From the final GRM, posterior trait estimates were generated and rescaled to create an IRT-based trust score. The distribution of these scores, shown in Fig 5, ranged from 0 to 10 with a mean of 5.27 (SE = 0.11, 95% CI: 5.07–5.48). Visual inspection indicated an approximately symmetric distribution with slight skew toward higher trust.

When examined across sociodemographic and socioeconomic characteristics, trust scores varied modestly, as summarized in Table 5 and fully reported in Table S1 of the S1 File.

**Table 5:** Survey-weighted mean trust in public health authorities refined IRT-based trust score (0-10).

Characteristic	Mean Trust Score (SE)	95% CI
Age		
20–34	5.19 (0.32)	4.57, 5.82
35–54	5.05 (0.14)	4.78, 5.33
55–74	5.41 (0.09)	5.23, 5.59
75+	6.04 (0.15)	5.74, 6.34
Race*		
White/Caucasian	5.21 (0.13)	4.97, 5.47
Black/African American	5.34 (0.23)	4.89, 5.79
Other (Unspecified)	5.39 (0.36)	4.67, 6.10
Community Size		
Large Urban/City (pop. ≥ 50K)	5.34 (0.12)	5.10, 5.58
Small Rural/Town (pop. < 50K)	5.06 (0.22)	4.62, 5.50
Education		
High School or Less	4.93 (0.20)	4.53, 5.32
Some College/Associate Degree	5.03 (0.16)	4.72, 5.34
Bachelor’s Degree	5.64 (0.16)	5.31, 5.96
Graduate Degree	6.07 (0.20)	5.67, 6.46
Occupation		
Professional/Managerial	5.50 (0.14)	5.24, 5.77
Healthcare	5.12 (0.24)	4.65, 5.58
Service	5.48 (0.23)	5.03, 5.93
Sales/Administration	5.37 (0.29)	4.80, 5.95
Manual/Skilled Labor	4.84 (0.19)	4.47, 5.21
Other/Mixed/Unemployed	4.63 (0.54)	3.56, 5.70
Family Income		
Low (<$25k)	5.31 (0.32)	4.69, 5.93
Lower–Middle ($25k–$49,999)	4.98 (0.23)	4.54, 5.43
Middle ($50k–$99,999)	5.30 (0.14)	5.02, 5.57
Upper–Middle ($100k–$199,999)	5.34 (0.25)	4.85, 5.83
High (≥$200k)	5.63 (0.27)	5.11, 6.15

* Complete demographic characteristics, including detailed race categories and additional subgroup estimates, are reported in Table S1 of the S1 File.

Patterns were most apparent across indicators of life stage and educational attainment, with higher average trust observed among older adults and respondents holding bachelor’s or graduate degrees. Differences by employment and occupation were also evident, with lower mean trust among respondents working in manual/skilled labor or unemployed relative to those in professional or managerial roles.

Place-based differences were smaller in magnitude but directionally consistent, with slightly higher average trust among urban residents compared to those living in rural or small-town communities. Across income categories, mean trust scores were generally similar, though respondents in the highest income group exhibited somewhat higher average trust. Differences by sex and nativity were also modest, with slightly higher trust among women and non-US-born respondents.

Across racial groups, descriptive differences were limited overall; however, mean trust was lowest among American Indian or Alaska Native respondents, though this estimate was based on a small subsample and should be interpreted cautiously. Higher average trust was observed among Asian or Pacific Islander respondents.

## Discussion

### Item response theory analysis

A key strength of this study lies in the use of IRT to refine measurement of trust in public health authorities. By assessing dimensionality and item-level performance of the TiPHA beneficence subscale, we identified meaningful variation in how individual items contributed to measurement precision and developed a refined, interpretable trust score.

The refined scale was most informative at moderate to high levels of trust, where measurement precision is greatest. This concentration of information aligns with the substantive importance of distinguishing among individuals who vary in their degree of confidence in public health authorities, while recognizing that measurement of very low trust remains less precise. This limitation is especially salient because the populations least trusting of public health authorities are often those most vulnerable in times of crisis [30,31], underscoring the need for future work to strengthen measurement at the lower end of the trust continuum.

These findings also highlight broader challenges inherent in analyzing Likert-type data. Individual Likert items are ordinal in nature: response categories have a rank order, but the distances between categories cannot be assumed equal [32]. Despite this, many studies treat such responses as interval-level data, calculating means and standard deviations or applying parametric tests, which risks misleading conclusions about the construct being measured [32]. Summed scores across multiple items can approximate interval properties, but this assumption is not guaranteed. Item-based psychometric methods such as IRT provide a principled solution. By modeling the probability of choosing each response category as a function of a respondent’s latent trait, IRT avoids the assumption of equal spacing between categories and leverages item-level parameters to rescale ordinal responses into an interpretable interval metric [33].

Beyond these empirical results, the application of IRT strengthens the methodological foundation for assessing institutional trust by enabling item-level evaluation of performance and measurement precision across the latent continuum. This approach supports both valid and efficient measurement and facilitates scale refinement. In this study, refinement from eight items to five reduced respondent burden, an important consideration for large national surveys where questionnaire length is tightly constrained.

Notably, the three items removed from the scale were negatively worded. Negatively phrased questions can introduce cognitive complexity, increase the risk of misinterpretation, and contribute to inconsistent response patterns, which may help explain their poor psychometric performance in this study [34]. This observation underscores the importance of careful item wording in scale development, as negative framing may reduce rather than enhance construct validity. At the same time, it raises challenges for comparability with prior studies that relied on raw Likert means, highlighting the need for transparent reporting of coding and scoring procedures.

Taken together, the refined five-item scale is well suited for use in population-based surveys and secondary data analyses where efficient, interpretable measurement of trust is required, particularly when distinguishing variation at moderate to high levels of trust.

### Application of refined trust scale

The observed demographic patterns provide additional face validity for the refined scale. Higher trust scores clustered among groups traditionally more embedded in institutional systems, including older adults, individuals with higher educational attainment, and those in professional or managerial occupations. These patterns are consistent with prior research suggesting that accumulated institutional exposure, familiarity with public systems, and access to educational resources may shape perceptions of public health authorities [35–37].

Across racial groups, differences in mean trust were generally modest. The lowest average trust was observed among American Indian or Alaska Native respondents. Although this estimate was based on a small subsample and should be interpreted cautiously, it aligns with a broader literature documenting historical and ongoing sources of institutional mistrust among Indigenous populations and other racial and ethnic minority groups [38,39]. Oversampling these populations in future research could help clarify trust patterns and assess whether additional items are needed to better capture lower levels of trust.

Differences in trust across income categories were limited, with largely overlapping confidence intervals, and non-US-born respondents exhibited slightly higher average trust than US-born respondents. These patterns differ from some prior studies that have linked lower socioeconomic status or immigrant identity to reduced institutional trust [40]. One possible explanation is that the refined measure captures a specific dimension of trust, namely perceived beneficence of public health authorities, rather than broader political or governmental trust. Trust grounded in perceived intent, fairness, and concern for public well-being may operate differently from trust rooted primarily in material resources or economic self-interest, particularly in public health settings.

Although descriptive differences across sociodemographic groups were generally modest, these findings motivate further multivariable and intersectional analyses to assess whether observed patterns persist after accounting for correlated social, economic, and structural factors. More broadly, they underscore the importance of using conceptually precise and psychometrically robust measures when examining trust, as distinct dimensions of trust may exhibit different relationships with socioeconomic position and social identity.

### Limitations

This study has limitations that should be considered when interpreting the findings. First, the refined TiPHA beneficence subscale demonstrated the greatest precision at moderate to high levels of trust, limiting the ability to distinguish among individuals with very low trust in public health authorities. In addition, prior research suggests that expressions of strong distrust may be understated in survey responses, as positively framed trust items and social desirability concerns can discourage respondents from endorsing very low levels of trust [41]. Together, these factors may contribute to floor effects that underestimate the extent of distrust.

Second, only the beneficence subscale of the TiPHA instrument was included in the Omnibus Wave 17 survey. While beneficence captures a central dimension of trust, the exclusion of competence items means that trust was not assessed in its full conceptual scope. Future work should incorporate both subscales to provide a more comprehensive evaluation of trust in public health authorities. Furthermore, data was collected within a single survey wave and context. Responses may vary across survey modes, time periods, or institutional settings, and future research should assess the stability of these parameters across contexts.

Third, the descriptive application of the refined trust score across sociodemographic groups was not intended to test explanatory or causal hypotheses. Observed subgroup differences should therefore be interpreted as illustrative of the scale’s behavior in a population-based sample rather than as evidence of underlying mechanisms. Moreover, this study did not formally assess differential item functioning across sociodemographic groups, an important area for future investigation.

Fourth, as with all IRT applications, the assumption of local independence must be considered. This assumption holds that once the latent trait is accounted for, responses to individual items are statistically independent. In practice, violations are common with multi-item instruments, and they can distort item parameters (e.g., inflating slopes or making thresholds appear more similar) and inflate information functions, giving a misleading impression of score precision [16].

Finally, although the ALP uses probability-based sampling to approximate national representativeness, participation and retention vary over time. Differential survey participation and panel attrition may therefore introduce bias and limit the generalizability of these findings.

## Conclusion

This study demonstrates the value of IRT for refining and validating measures of trust in public health authorities. By applying IRT to the TiPHA beneficence subscale in a nationally representative U.S. survey, we identified and removed poorly performing items, yielding a concise five-item scale with strong reliability and discrimination. The refined scale was most precise at moderate to high trust levels and provides a psychometrically rigorous alternative to traditional mean-based scoring of Likert data. By demonstrating how the refined score behaves across key sociodemographic characteristics, this study also illustrates its utility for population-based descriptive analyses and for informing future research on trust disparities.

Although challenges remain, this work establishes a stronger foundation for measuring trust in ways that are both efficient and valid. Future research should apply these methods to assess trust across time and contexts, supporting longitudinal comparison and the continued development of robust measurement tools for public health research.

## Supporting information

S1 File This file contains the following supplementary tables. Table S1. Characteristics of Survey Respondents, Including Survey-Weighted Mean Trust Scores Using a Refined 5-Item TiPHA Beneficence Subscale (N = 2,034); Table S2. Item-Level Missingness for the TiPHA Beneficence Items; and Table S3. Polychoric Correlation Matrix for the 8-Item TiPHA Beneficence Subscale.
(DOCX)
