# Supplementary material for: Measuring trust in public health authorities in the United States: An item response theory analysis
Source: PLoS One. 2026 Aug 6;21(8):e0355498. doi: 10.1371/journal.pone.0355498 (PMC13446734; doi:10.1371/journal.pone.0355498)
Supplement: Supplementary file 1 [file pone.0355498.s001.docx]

**Appendix**

**Table S1.** Characteristics of Survey Respondents, Including Survey-Weighted Mean Trust Scores Using a Refined 5-Item TiPHA Beneficence Subscale (N = 2,034)

| **Characteristic** | **n (%)** | **Trust Score; 0-10 (SE)** | **95% CI** |
| --- | --- | --- | --- |
| Sex | | | |
| Female | 1,041 (51.2%) | 5.40 (0.12) | 5.17, 5.64 |
| Male | 993 (48.8%) | 5.14 (0.17) | 4.80, 5.47 |
| Age | | | |
| 20 - 34 | 559 (27.5%) | 5.19 (0.32) | 4.57, 5.82 |
| 35 - 54 | 690 (33.9%) | 5.05 (0.14) | 4.78, 5.33 |
| 55 - 74 | 645 (31.7%) | 5.41 (0.09) | 5.23, 5.59 |
| 75+ | 140 (6.9%) | 6.04 (0.15) | 5.74, 6.34 |
| Race | | | |
| White/Caucasian | 1,530 (75.2%) | 5.21 (0.13) | 4.97, 5.47 |
| Black/African American | 256 (12.6%) | 5.34 (0.23) | 4.89, 5.79 |
| American Indian/Alaska Native | 24 (1.2%) | 4.71 (0.57) | 3.59, 5.83 |
| Asian or Pacific Islander | 61 (3.0%) | 6.34 (0.23) | 5.88, 6.80 |
| Other (Unspecified) | 163 (8.0%) | 5.39 (0.36) | 4.67, 6.10 |
| Ethnicity | | | |
| Hispanic/Latinx | 384 (18.9%) | 4.86 (0.28) | 4.32, 5.41 |
| Non-Hispanic/Latinx | 1,650 (81.1%) | 5.37 (0.12) | 5.14, 5.60 |
| Nativity | | | |
| US-Born | 1,837 (90.3%) | 5.22 (0.11) | 5.00, 5.44 |
| Non-US-Born | 197 (9.7%) | 5.81 (0.30) | 5.22, 6.39 |
| Birth Region | | | |
| Northeast | 476 (23.4%) | 5.20 (0.20) | 4.81, 5.58 |
| Midwest | 407 (20.0%) | 5.57 (0.16) | 5.26, 5.89 |
| South | 747 (36.7%) | 5.09 (0.23) | 4.64, 5.55 |
| West | 404 (19.9%) | 5.14 (0.18) | 4.78, 5.50 |
| Residence Region | | | |
| Northeast | 417 (20.5%) | 5.48 ( 0.22) | 5.06, 5.90 |
| Midwest | 323 (15.9%) | 5.67 (0.20) | 5.28, 6.05 |
| South | 840 (41.3%) | 5.05 (0.19) | 4.68, 5.43 |
| West | 454 (22.4%) | 5.21 (0.15) | 4.92, 5.50 |
| Community Size | | | |
| Large Urban/City (pop. ≥50K) | 1,540 (75.7%) | 5.34 (0.12) | 5.10, 5.58 |
| Small Rural/Town (pop. <50K) | 494 (24.3%) | 5.06 (0.22) | 4.62, 5.50 |
| Education | | | |
| High School or Less | 777 (38.2%) | 4.93 (0.20) | 4.53, 5.32 |
| Some College/Associate Degree | 535 (26.3%) | 5.03 (0.16) | 4.72, 5.34 |
| Bachelor’s Degree | 399 (19.6%) | 5.64 (0.16) | 5.31, 5.96 |
| Graduate Degree | 323 (15.9%) | 6.07 (0.20) | 5.67, 6.46 |
| Employment Status | | | |
| Working | 916 (45.0%) | 5.23 (0.14) | 4.96, 5.51 |
| Retired | 682 (33.5%) | 5.76 (0.14) | 5.50, 6.03 |
| Disabled | 92 (4.5%) | 5.31 (0.23) | 4.85, 5.77 |
| Homemaker | 51 (2.5%) | 5.16 (0.45) | 4.27, 6.04 |
| Laid Off | 9 (0.4%) | 5.74 (0.95) | 3.88, 7.60 |
| Unemployed | 39 (1.9%) | 5.26 (0.65) | 3.98, 6.55 |
| Other (Unspecified) | 28 (1.4%) | 4.34 (0.46) | 3.43, 5.24 |
| Multiple Statuses | 217 (10.7%) | 5.21 (0.23) | 4.76, 5.65 |
| Occupation | | | |
| Professional/Managerial | 752 (37.0%) | 5.50 (0.14) | 5.24, 5.77 |
| Healthcare | 201 (9.9%) | 5.12 (0.24) | 4.65, 5.58 |
| Service | 256 (12.6%) | 5.48 (0.23) | 5.03, 5.93 |
| Sales/Administration | 364 (17.9%) | 5.37 (0.29) | 4.80, 5.95 |
| Manual/Skilled Labor | 323 (15.9%) | 4.84 (0.19) | 4.47, 5.21 |
| Other/Mixed/Unemployed | 138 (6.8%) | 4.63 (0.54) | 3.56, 5.70 |
| Family Income | | | |
| Low (<$25k) | 252 (12.4%) | 5.31 (0.32) | 4.69, 5.93 |
| Lower - Middle ($25k - $49,999) | 399 (19.6%) | 4.98 (0.23) | 4.54, 5.43 |
| Middle ($50k - $99,999) | 635 (31.2%) | 5.30 (0.14) | 5.02, 5.57 |
| Upper - Middle ($100k - $199,999) | 618 (30.4%) | 5.34 (0.25) | 4.85, 5.83 |
| High (≥$200k) | 130 (6.4%) | 5.63 (0.27) | 5.11, 6.15 |
| Housing Tenure | | | |
| Own with Mortgage | 867 (42.7%) | 5.36 (0.19) | 4.99, 5.72 |
| Own without Mortgage | 470 (23.1%) | 5.22 (0.18) | 4.86, 5.58 |
| Rent | 683 (33.6%) | 5.19 (0.18) | 4.84, 5.54 |
| No Rent/Other (Unspecified) | 14 (0.7%) | 5.78 (0.51) | 4.78, 6.78 |
| Housing Type | | | |
| Detached Home | 1,255 (61.7%) | 5.28 (0.14) | 5.01, 5.56 |
| Attached Home/Small Apartment | 283 (13.9%) | 5.30 (0.23) | 4.84, 5.76 |
| Medium Apartment | 222 (10.9%) | 5.73 (0.20) | 5.34, 6.11 |
| Large Apartment | 150 (7.4%) | 5.76 (0.29) | 5.20, 6.31 |
| Other (mobile home, boat, RV, van, etc.) | 124 (6.2%) | 3.76 (0.48) | 2.81, 4.71 |
| Household Size | | | |
| Lives Alone | 307 (15.1%) | 5.43 (0.22) | 5.00, 5.86 |
| Two-Person Household | 720 (35.4%) | 5.55 (0.12) | 5.32, 5.77 |
| Three or Four-Person Household | 834 (41.0%) | 5.12 (0.20) | 4.74, 5.51 |
| ≥ Five-Person Household | 173 (8.6%) | 4.60 (0.41) | 3.79, 5.41 |
| Insurance Type | | | |
| Private Insurance | 1,341 (65.9%) | 5.27 (0.14) | 4.99, 5.54 |
| Public Insurance | 590 (29.0%) | 5.35 (0.16) | 5.03, 5.68 |
| Other/Uninsured | 103 (5.2%) | 4.93 (0.39) | 4.18, 5.69 |

*Note:* Trust scores are derived from a refined 5-item IRT-based Trust in Public Health Authorities (TiPHA) Scale (0-10), with higher scores indicating greater trust. Estimates are survey-weighted. Standard errors (SEs) and 95% confidence intervals (CIs) are shown. Estimates for categories with small sample sizes should be interpreted with caution.

**Table S2.** Item-Level Missingness (Not Applicable/Don’t Know) for TiPHA Beneficence Items

| **Item** | **Unweighted n (%) Missing** | **Survey-Weighted % Missing** |
| --- | --- | --- |
| 1. They do everything they should to protect the health of the public. | 32 (1.57%) | 1.40% |
| 2. They keep trying the same things to help the public, even when they don’t work very well.* | 72 (3.54%) | 3.57% |
| 3. They base recommendations on the best available science. | 38 (1.87%) | 1.80% |
| 4. They are concerned about all people, without caring about who has more or less money. | 36 (1.77%) | 1.89% |
| 5. They are concerned about some racial and ethnic groups more than others.* | 74 (3.64%) | 3.72% |
| 6. They accurately inform the public of both health risks and benefits of medicines. | 29 (1.43%) | 1.50% |
| 7. They make unhelpful recommendations.* | 46 (2.26%) | 1.97% |
| 8. They believe in what they recommend for the public. | 79 (3.88%) | 2.97% |

* Negatively worded items were reverse coded prior to analysis to ensure comparability.

*Note:* Percentages are calculated using N = 2,034 respondents. “Missing” reflects responses coded as “Not Applicable/Don’t know.”

**Table S3.** Polychoric Correlation Matrix of TiPHA Beneficence Items (Full 8-Item Set)

| **Item** | **Q1** | **Q2*** | **Q3** | **Q4** | **Q5*** | **Q6** | **Q7*** | **Q8** |
| --- | --- | --- | --- | --- | --- | --- | --- | --- |
| **Q1** | 1.00 | 0.21 | 0.78 | 0.74 | 0.18 | 0.75 | 0.28 | 0.66 |
| **Q2*** | - | 1.00 | 0.20 | 0.18 | 0.42 | 0.18 | 0.43 | 0.23 |
| **Q3** | - | - | 1.00 | 0.71 | 0.15 | 0.78 | 0.35 | 0.72 |
| **Q4** | - | - | - | 1.00 | 0.31 | 0.70 | 0.27 | 0.68 |
| **Q5*** | - | - | - | - | 1.00 | 0.15 | 0.32 | 0.19 |
| **Q6** | - | - | - | - | - | 1.00 | 0.34 | 0.68 |
| **Q7*** | - | - | - | - | - | - | 1.00 | 0.27 |
| **Q8** | - | - | - | - | - | - | - | 1.00 |

*Note:* Correlations are polychoric, estimated using survey weights. All associations are positive and in the expected direction. Correlations among positively worded items are moderate to strong (≈ 0.65-0.78), while reverse-coded items show weaker relationships (≈ 0.15-0.43), supporting unidimensionality of the beneficence construct. * = reverse-coded item.
